# “Shadow” OSCE examiner. A cross-sectional study comparing the “shadow” examiner with the original OSCE examiner format

**DOI:** 10.6061/clinics/2019/e1502

**Published:** 2019-11-04

**Authors:** Marcelo Arlindo Vasconcelos Rodrigues, Rodrigo Diaz Olmos, Célia Maria Kira, Paulo Andrade Lotufo, Itamar Souza Santos, Iolanda de Fátima Lopes Calvo Tibério

**Affiliations:** I Divisao de Clinica Medica, Hospital Universitario, Universidade de Sao Paulo, Sao Paulo, SP, BR; II Departamento de Clinica Medica, Faculdade de Medicina FMUSP, Universidade de Sao Paulo, Sao Paulo, SP, BR

**Keywords:** OSCE, Feedback, Assessment, Examiner, Formative

## Abstract

**OBJECTIVES:**

Feedback is a powerful learning tool, but a lack of appropriate feedback is a very common complaint from learners to teachers. To improve opportunities for feedback on objective structured clinical examinations (OSCEs), a modified examiner role, termed the “shadow” examiner, was tested. This study aims to present and analyze comparisons between the “shadow” examiner and the original OSCE examiner format.

**METHODS:**

In 2011, experiments were carried out with modifications to the examiner’s role to define the “shadow” examiner format. From February 2012 to May 2014, research was conducted with 415 6^th^-year medical students. Of these students, 316 were randomly assigned to assessments by both “shadow” and “fixed” examiners. Pearson correlation analysis with linear regression, Student’s t-tests and Bland-Altman plots were the statistical methods used to compare the assessment modes. To strengthen the analysis, checklist items were classified by domain.

**RESULTS:**

High correlations between the “shadow” and “fixed” examiners’ global scores were observed. The results of the analysis of specific domains demonstrated higher correlations for cognitive scores and lower correlations for affective scores. No statistically significant differences between the mean examiner global scores were found. The Bland-Altman analysis showed that the “shadow” examiners’ affective scores were significantly higher than those of the “fixed” examiners, but the magnitude of this difference was small.

**CONCLUSION:**

The modified examiner role did not lead to any important bias in the students’ scores compared with the original OSCE examiner format. This new strategy may provide important insights for formative assessments of clinical performance.

## INTRODUCTION

The objective structured clinical examination (OSCE) is one of the most popular forms of clinical skills assessment. In OSCEs, students rotate sequentially through clinical scenarios (stations) and are required to perform a variety of tasks. The time to complete each station may vary, but 5 to 15 minutes is the most typical. Assessment of the ability to perform clinical procedures and examination in a structured way is a great advantage of this method (1,2). The OSCE is a performance assessment and is focused on what students can do (by showing) rather than what they know or know how to do (3,4).

In addition to allowing for assessment, an OSCE may be a strategic moment to provide adequate feedback (5-9). Feedback in clinical education can be defined as &quot;specific information about the comparison between a trainee's observed performance and a standard, given with the intent to improve the trainee's performance&quot; (10). It is a powerful learning tool and an essential element of the educational process for clinical training. Feedback must be structured to allow timely delivery and to address personal performance to enable examiners to work as student’s allies based on first-hand data. Clearly, delivering individualized and targeted feedback immediately after an assessment is considered to be a high-impact educational strategy. Feedback should be provided for all students, especially those who need more feedback than others (11-13).

However, there are some challenges in providing feedback after an OSCE. Typically, examiners observe and score students at one specific station (“fixed” examiners). Therefore, a single examiner does not have access to the entire performance of the student, and feedback can be provided only by a group of examiners or by proxies. Thus, we seek to modify the OSCE assessment format, particularly in relation to the role of examiners, to provide better feedback.

In this regard, we propose the role of a “shadow” examiner who accompanies the student through each of the stations; only after the student has completed all OSCE stations can the examiner provide structured, targeted and individualized feedback. However, the possibility of assessment bias by the “shadow” examiner needs to be excluded to consider this strategic modification of the OSCE assessment to be a reliable methodology.

In this paper, we aimed to compare the scores attributed by “shadow” and “fixed” examiners to search for potential assessment bias arising from this new examiner strategy for the OSCE.

## METHODS

This study is descriptive, analytical, and cross-sectional and takes a quantitative approach to examining OSCE assessment by comparing scores provided by “shadow” and “fixed” examiners for final-year medical students of medicine from February 2012 to May 2014.

### Study setting

This study was conducted at the Hospital Universitário da Universidade de São Paulo (HU-USP), a community teaching hospital located in Butantã, São Paulo, Brazil. OSCEs were administered from February 2012 to May 2014.

The students being assessed were in their last year of the Faculdade de Medicina da Universidade de São Paulo (FMUSP) medical program. All Brazilian medical schools have a six-year curriculum. In the FMUSP, the internship rotations occur during the final two years of undergraduate education and involve supervised hands-on training, mainly in two university hospitals, the HU-USP and a tertiary hospital, Hospital das Clínicas da FMUSP (HC-FMUSP). OSCEs have been part of the internship assessment in the FMUSP since 2002. Currently, this tool is incorporated in almost all internship rotations (14).

At the HU-USP, the Internal Medicine Internship (IMI) rotation (a 7-week program) occurs in the last year of training. The rotation involves annual supervised and hands-on training of 175 students. Students perform supervised clinical activities and simulations in four areas: the emergency room, clinical ward, intensive care unit and ambulatory outpatient clinics. Students’ assessments in the IMI rotation include a written test, overall ratings by supervisors and an OSCE using standardized patients and simulation mannequins.

### “Shadow” examiners for the OSCE of the HU-USP IMI rotation

Aiming to improve formative feedback, we developed a different OSCE examiner strategy. This new strategy maintains the essential structure of the OSCE. In varying clinical scenarios, students must perform specific tasks at a series of different stations within a given time. The OSCE is a performance assessment, and all components of the competencies are assessed in a planned way. Simulated patients, video and sound recordings, and simulators are used to simulate practical clinical scenarios. The examiner is trained on the expected tasks and completes a checklist, reflecting the student’s actual performance (1,2,5,6).

However, in this new form, the same examiner accompanies the student through all stations (“shadow” examiner). The &quot;shadow” examiner is responsible for the completion of all checklists for a particular student. At the end of the OSCE, the “shadow” examiner provides structured, individualized, and targeted feedback to the student.

In 2011, 25 different stations with emergency room, clinical ward, intensive care unit and ambulatory clinic scenarios were created. These stations were completed by 173 6^th^ year students in the IMI of the HU-USP, and 36 medical professionals from the HU-USP staff were trained as “shadow” examiners. Discussions, face-to-face interviews and written forums involving students and the staff responsible for administering the OSCE, including the authors of this paper, were used to define the ideal characteristics of the “shadow” examiner. The time required for the tasks and stations, feedback times, the adequacy of each task or scenario in representing a discipline’s objectives, the creation of mannequins appropriate for the necessary simulation and available funds, and the best process for training examiners were defined in 2011. These experiences were important for defining all characteristics of the “shadow” examiner who would assess students during the OSCE.

The training of the “shadow” examiners started one week before each OSCE. First, the examiners were individually trained by the OSCE coordinator. This training included written and verbal information about the scenario content, the tasks and checklists of all stations and specific guidance about how to provide feedback to the students. Second, the week before the OSCE, the examiners studied all materials, were on hand to answer questions from students and clarified their own doubts via email, telephone and/or in person. Third, on the day of the OSCE, one hour before beginning, there was a meeting with all examiners, and all station objectives and the method of provision of adequate feedback were reinforced. Fourth, all examiners were directed to the area where the stations were assembled for simulations and final considerations.

The same training structure was used for the “fixed” examiners as for the “shadow” examiner, except for the training on how to provide feedback. The “fixed” examiners received their training only at the specific station that they were assigned to assess.

The “shadow” examiners received specific training about how to provide structured, individualized and targeted feedback. Structured feedback had a preset duration (40% of the total station time) and involved all stations and tasks performed by the student. Students had access to the checklists and the assessment of their performance to optimize the discussion about their potential for improvement. Students received individualized feedback only at the end of the test. Only the student and the “shadow” examiner remained at the last station during the feedback time. All feedback provided to the student was targeted to improve the competencies and abilities that were the focus of the OSCE.

### Study design

This study compares data from a suitable sample of OSCE stations in which students were evaluated by both “shadow” and “fixed” examiners. These stations were not selected using any specific criteria and were subject to the availability of a sufficient number of trained examiners at a given OSCE. During the study period, there were 594 OSCE station applications. In each session, a student was assessed by both &quot;shadow&quot; and &quot;fixed&quot; examiners.

### Checklist classification

All checklist items were classified based on the 3 primary domains of Bloom’s taxonomy (affective, psychomotor and cognitive). The classification of the items according to this taxonomy was based on learning objectives using the following definitions: (a) affective abilities – these items involved abilities related to feelings and attitudes and were categorized in relation to the development of emotional and affective areas, including behavior, attitudes, responsibility, respect, emotion and values; (b) psychomotor abilities – these items were related to specific motor skills that involve reflexes and perception and that require muscular skill, the manipulation of material and objects and fine movements, such as performing a medical history and/or physical exam; and (c) cognitive abilities – these items were related to learning, from the mastering of theoretical knowledge to the intellectual aspect of a skill or attitude, and they required remembering or reproducing something that was learned previously (15-17).

Two authors (RDO, CMK) classified the OSCE checklist items according to these domains. When there were disagreements in the classification (78/451 items; 17.3%), the three other authors (MAVR, ISS, IFCT) finalized their classification by consensus. However, we observed that 15 (3.3%) checklist items evaluated more than one ability, and there was no consensus for these items. These items were not analyzed for the domain-specific scores but remained in the global scores.

### Ethical considerations

This study was approved by the FMUSP Institutional Review Board.

### Statistical analysis

Categorical variables are presented as counts and proportions, and quantitative variables are presented as the means and standard deviations. Domain-specific scores (affective, psychomotor and cognitive) are the sum of all items with the same classification. Global scores are the sum of all checklist items for a station application. We evaluated the interobserver correlation between “shadow” and “fixed” examiners’ affective, psychomotor, cognitive and global scores using Pearson correlation analysis. We compared the mean score differences for the “shadow” and “fixed” examiners’ scores using Student’s t-tests and analyzed the distribution of differences using Bland-Altman plots.

We used R software version 3.2.0 for the analyses. The significance level was set at 0.05.

## RESULTS

Table 1 shows the descriptive data for our study sample. During the study period, 594 OSCE station applications (10,833 OSCE checklist items) were scored by both “shadow” and “fixed” examiners. We identified 451 OSCE checklist items for the stations, as shown in Table 1. Of the 451 items, 39 (8.6%) were classified as assessing affective abilities, 169 (37.4%) were classified as assessing psychomotor abilities, 228 (50.6%) were classified as assessing cognitive abilities, and 15 (3.3%) were not classified.

Pearson’s correlation coefficients between the “shadow” and “fixed” examiners’ affective, psychomotor, cognitive and global scores are shown in Table 2. We observed high correlations between the “shadow” and “fixed” examiners’ global scores (*r*=0.87; 95% *CI*: 0.85 − 0.89). Analyzing the specific domains, we found higher correlations for the cognitive scores (*r*=0.86; 95% *CI*: 0.84 − 0.88) and lower correlations for the affective scores (*r*=0.72; 95% *CI*: 0.67 − 0.76). We observed strong correlations (Pearson’s correlation coefficient greater than 0.70) (18) for all specific domains.

Table 3 shows the comparison between the mean “shadow” and “fixed” examiners’ scores. We found no significant differences between the mean “shadow” and “fixed” examiners’ global scores, expressed on a scale from zero to 100 (mean difference, +0.6; 95% *CI*: -0.1 to +1.2; *p*=0.070) using Student’s t-tests. For the affective scores, we found that the “shadow” examiners’ mean scores were significantly higher than those of the “fixed” examiners, but the magnitude of this difference was small (+3.0; 95% *CI*: +0.2 to +5.8; *p*=0.035). No other significant differences were found for the psychomotor and cognitive domains.

**Figure 1:**
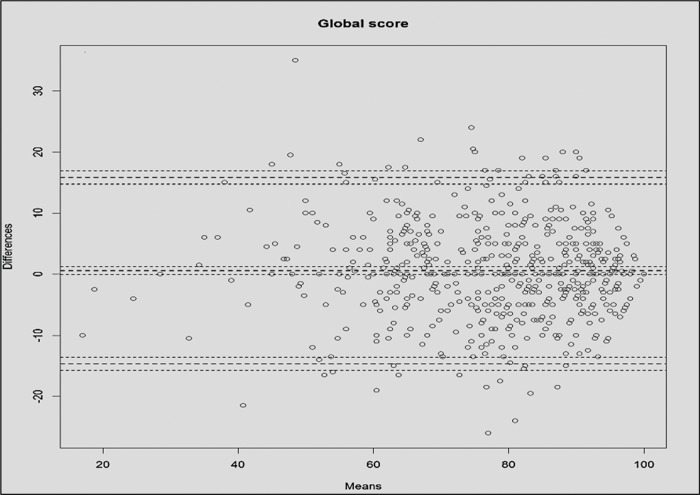
Bland-Altman plot to compare the “shadow” and “fixed” examiners’ global scores*. *Global score = sum of all checklist items in a station application.

The Bland-Altman plot for the comparison between the “shadow” and “fixed” examiners’ global scores is shown in Figure 1. The pattern of differences was similar across all of the score ranges, except for very high mean scores (near 100 points), when, as expected, the differences between the “shadow” and “fixed” examiners’ scores were very small.

## DISCUSSION

We described a new strategy for OSCE assessment with enhanced opportunities for feedback. The global and domain-specific scores attributed by “shadow” and “fixed” examiners had good correlations. Except for the affective scores, for which we observed a small (3%) bias towards higher scores from the “shadow” examiners, the assessments provided by “shadow” and “fixed’ examiners were similar.

Since the publication of the first OSCE studies, the potential of the OSCE for providing feedback during in-course assessment that focuses on different components of clinical competence has been shown (5). The OSCE is a powerful tool that can determine the preparation of the medical professional by creating situations that reinforce medical professional performance. The existence of the OSCE induces a series of professional behaviors in medical schools and in clinical practice (19). In this context, providing feedback may be valuable. Providing students with feedback enhances learning, and feedback provided by an OSCE can lead to better student performance (20,21).

However, as previously stated, an educational impact of feedback in OSCE assessments is not easily achieved (20,22,23). Moreover, feedback is often not provided or is provided in an inadequate way (11), which is additional evidence of how difficult providing feedback for OSCEs may be. The “shadow” examiner format was created to provide structured, individualized and targeted feedback to students that is focused on the formative aspect of the OSCE. The “shadow” examiner format is intended to be an optimized formative assessment tool that maintains the valid and reliable characteristics of the conventional OSCE examiner format. To achieve this goal, we propose a modification to the examiner’s role during the assessment.

There are various forms in which to provide individual or group feedback during or after an OSCE assessment. Some possibilities for providing individualized feedback are incorporation the feedback at the end or in the final minutes of each station during the examination (21,24,25). However, we suggest some advantages of providing feedback at the end of the entire OSCE assessment through a “shadow” examiner who has been assessing the student at all stations. First, giving feedback to the student before all stations have been completed may interfere (positively or negatively) with the progress of the test. Second, having the examiner follow the student through all stations, similar to a shadow, offers the advantage of the examiner having a more comprehensive view of the student to provide more detailed feedback. It is as if the examiner is observing a “film” of the student’s performance, not only a “picture”. In addition, the provision of feedback immediately at the end of the entire OSCE is still timely, as students still remember the station content.

Considering that the study was the first application of this new strategy, we also listened to the students' opinions. According to questionnaires completed at the end of each OSCE during the period from February 11, 2012 to May 31, 2014, all 415 6^th^-year students who participated in the OSCE assessments (data not shown) stated that the feedback provided by the “shadow” examiners contributed to their skill training. More than 90% of the students agreed that this form of feedback was more effective than other feedback strategies used in OSCE assessments in the medical program (data not shown).

Our analyses did not rely on correlation measurements alone to assess the hypothesis that the “shadow” examiners’ assessments would not be different from those of the “fixed” examiners. Correlation coefficients may be high even in the presence of significant bias or poor agreement between two measurements (26-28). Beyond adopting the widely used t-tests to compare the global and ability-specific means, we also present our results as Bland-Altman plots. These plots enable the determination whether significant bias exists between two measurements and whether the differences between scores are homogeneous across the measurement range (29). There was no significant bias for most of the scores, given that the confidence interval for the mean difference included null (zero difference). The only exception were the affective scores, which presented a small but significant bias towards higher scores for the “shadow” examiners’ assessments compared to the “fixed” examiners’ assessments. Differences in the global and ability-specific scores were found to be similar because the points on the plots are distributed in an unspecific, cloud-like pattern.

To analyze the small bias towards higher affective skill scores in the assessments by the “shadow” examiners compared to those by the “fixed” examiners, we separated the 39 items that were classified as affective abilities to analyze the agreement between the item scores lower than 70% given by “shadow” and “fixed” examiners. These items included greeting patients, requesting patient authorization for procedures, qualifying himself or herself as a student or team member, and communicating a certain procedure in language appropriate for the patient. These items are related to communication skills and empathy. A closer relationship between the student and the “shadow” examiner resulting from the examiner accompanying the student through all stations might lead to a biased assessment.

There are some drawbacks in the implementation of the “shadow” examiner format that require attention. Because “shadow” examiners follow the students through all stations, training these examiners is a more complex task. “Shadow” examiners must be prepared to perform at different stations. This characteristic can hinder the retention of necessary information because the examiner must know an increased amount of content. In addition, the feedback time increases the total test time. Through our study of the topic for one year in 2011, we found that allotting over 40% of the total time at all the stations to perform structured, individualized and targeted feedback was ideal.

We were able to study multiple OSCEs with a moderately high number of medical students. “Shadow” and “fixed” examiners assessed students independently. In addition, two doctors with experience in medical education performed the checklist item classification independently. However, our study must be interpreted in light of its limitations. We analyzed data from a single medical program in Brazil. We cannot conclude that the “shadow” examiner strategy would be feasible and reliable in other scenarios. The “shadow” examiner was applied in a single specialty (internal medicine). Although we expect that the absence of significant bias may not exist in other medical areas, this question still remains to be evaluated. In our study, all examiners were experienced professionals. Given that the examiners must be trained for multiple stations and provide comprehensive feedback, our results may not extend to scenarios in which examiners do not have such backgrounds.

## CONCLUSIONS

We described a new strategy for OSCE assessment, the “shadow” examiner, that is focused on providing enhanced formative feedback through a modification of the examiner’s role. These modifications did not lead to any important bias in students’ scores. This new strategy may provide important insights for formative assessment in clinical performance.

## AUTHOR CONTRIBUTIONS

Rodrigues MAV, Santos IS and Tibério IFLC developed the idea of the “shadow” examiner, they also developed the methodology, acquired the necessary data, organized, analyzed and interpreted the data, and drafted the entire manuscript. Olmos RD and Kira CM critically analyzed the data, classified the checklist items and contributed to writing parts of the manuscript. Lotufo PA contributed to organizing and implementing the OSCE program and provided critical analysis of the manuscript. All authors read and approved the final version of the manuscript.

## Figures and Tables

**Table 1:** Study sample descriptions.

	n
Students evaluated	316
OSCE scenarios	
Outpatient, scheduled consultation	5 (20.0%)
Outpatient, emergency consultation	5 (20.0%)
Inpatient, non-ICU	8 (32.0%)
Inpatient, ICU	7 (28.0%)
Total	25 (100%)
Student assessments by OSCE scenario	
Outpatient, scheduled consultation	143 (24.1%)
Outpatient, emergency consultation	110 (18.5%)
Inpatient, non-ICU	187 (31.5%)
Inpatient, ICU	154 (25.9%)
Total	594 (100%)
Checklist item classification / Number of applications	N of items
Affective 919 (8.5%)	39 (8.6%)
Psychomotor 3,910 (36.1%)	169 (37.4%)
Cognitive 5,647 (52.1%)	228 (50.6%)
Not Classified 357 (3.3%)	15 (3.3%)
Total 10,833 (100%)	451 (100%)

OSCE = Objective Structured Clinical Examination; ICU = Intensive Care Unit

**Table 2:** Pearson’s correlations (95% confidence intervals) for the “shadow” and “fixed” examiners’ scores.

	Pearson’s correlation coefficient “*r*”	*p*
Affective	0.72 (0.67 - 0.76)	<0.001
Psychomotor	0.75 (0.72 - 0.79)	<0.001
Cognitive	0.86 (0.84 - 0.88)	<0.001
Global*	0.87 (0.85 - 0.89)	<0.001

* Global = sum of all checklist items for a station application

**Table 3:** Student’s t-tests to compare the “shadow” and “fixed” examiners’ mean scores.

	“Shadow” examiners	“Fixed” examiners	Mean difference	*p*
Affective	70.4 ± 38.7	67.4 ± 40.0	+3.0 (+0.2 to +5.8)	0.035
Psychomotor	83.5 ± 21.7	83.6 ± 22.6	-0.1 (-1.4 to +1.2)	0.865
Cognitive	77.2 ± 18.2	76.5 ± 18.2	+0.7 (-0.1 to +1.5)	0.070
Global*	78.9 ± 15.2	78.3 ± 15.5	+0.6 (-0.1 to +1.2)	0.070

* Global = sum of all checklist items for a station application
